# Supplementary figures and images for: Stem cell culture conditions affect in vitro differentiation potential and mouse gastruloid formation
Source: PLoS One. 2025 Mar 26;20(3):e0317309. doi: 10.1371/journal.pone.0317309 (PMC11940422; doi:10.1371/journal.pone.0317309)

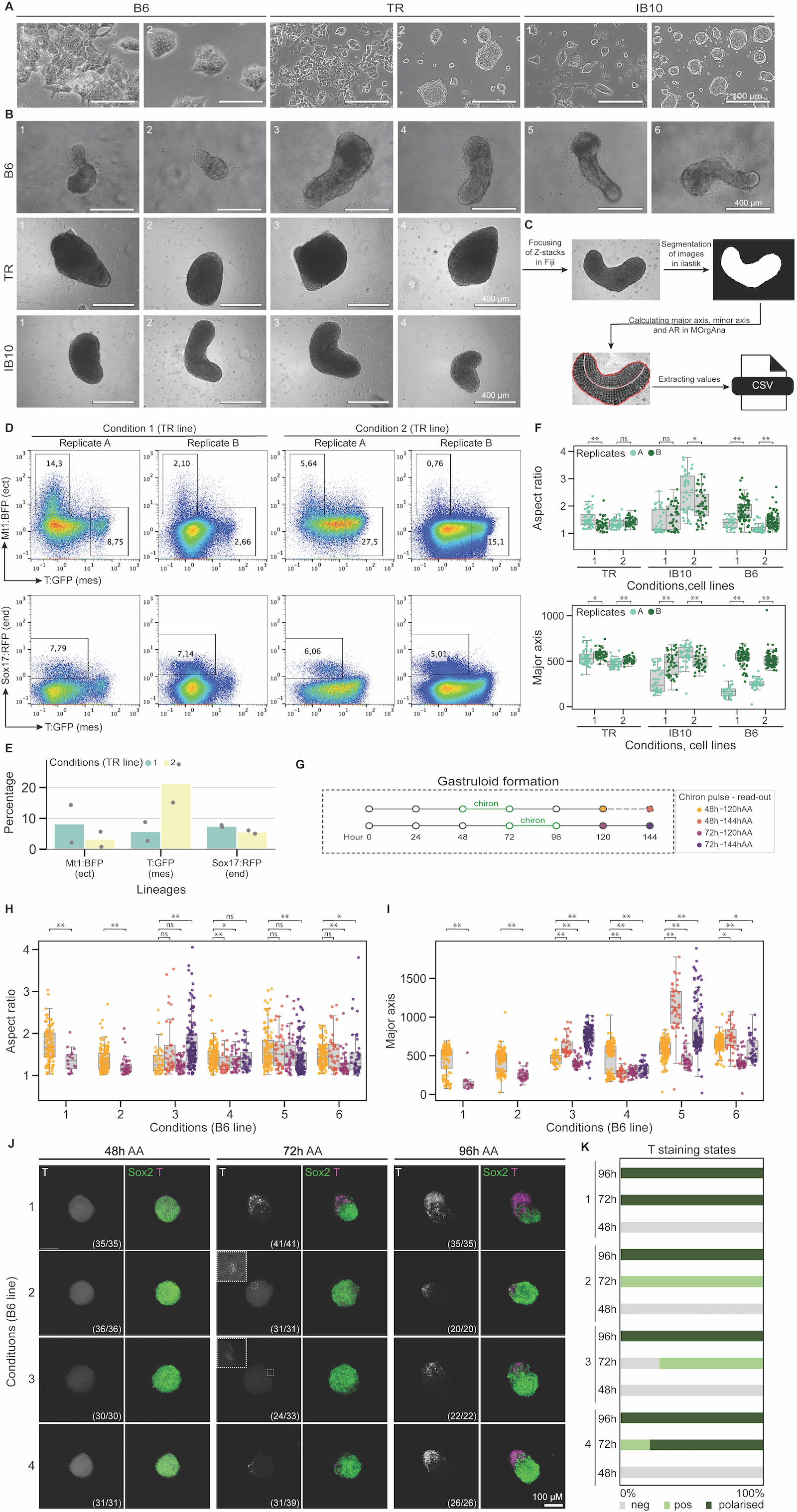

Supplement: S1 Fig — (A) Bright-field images showing mESC morphologies of condition 1 (serum) and condition 2 (2i) pre-cultures for all three cell lines. (B) Examples of gastruloid morphologies generated from the different pre-culture conditions for the three different cell lines. (C) Workflow for quantification of major axis length, minor axis length and aspect ratio of gastruloids. 96-well plates with gastruloids were imaged, and images of gastruloids were loaded into Fiji. Images were segmented using Ilastik, MOrgAna [36] was used to calculate the major and minor axis length. The aspect ratio was calculated by dividing the major axis length by the minor axis length of each gastruloid. (D-E) FACS plots (D) and quantification (E) of gastruloids formed using the TR line for condition 1 (serum) and condition 2 (2i). Mt1:BFP marks ectoderm (ect), T:GFP mesoderm (mes), and Sox17:RFP endoderm (end). (F) Aspect ratio and major axis length of gastruloids after condition 1 (serum) and condition 2 (2i) pre-cultures from all three cell lines, separated by replicate. Statistical significance between replicates A and B was calculated with an independent t-test. (G) Schematic overview of chiron pulse and read-out optimisation. For each pre-culture condition, the effect of conventional timing of the chiron pulse (48-72h AA: light orange, dark orange) and a delayed chiron pulse (72-96h AA: light purple, dark purple) on gastruloid formation were assessed. Gastruloid formation was assessed at 120 h AA and 144 h, and the aspect ratio (H) and major axis length (I) were calculated. Statistical significance of the differences in chiron pulse and read-out within conditions were calculated with Mann-Whitney U test with Bonferroni multiple testing correction. (J) Sox2 (green) and T (magenta) stainings and quantification (K) of pre-cultured gastruloids at 48h, 72h, and 96h AA. ES cell cultures were pre-treated with ESLIF (c1) or 2i from 96h (c2), 48h (c3) or 24h (c4) prior to the start of the gastruloid protoc [file pone.0317309.s001.tif]

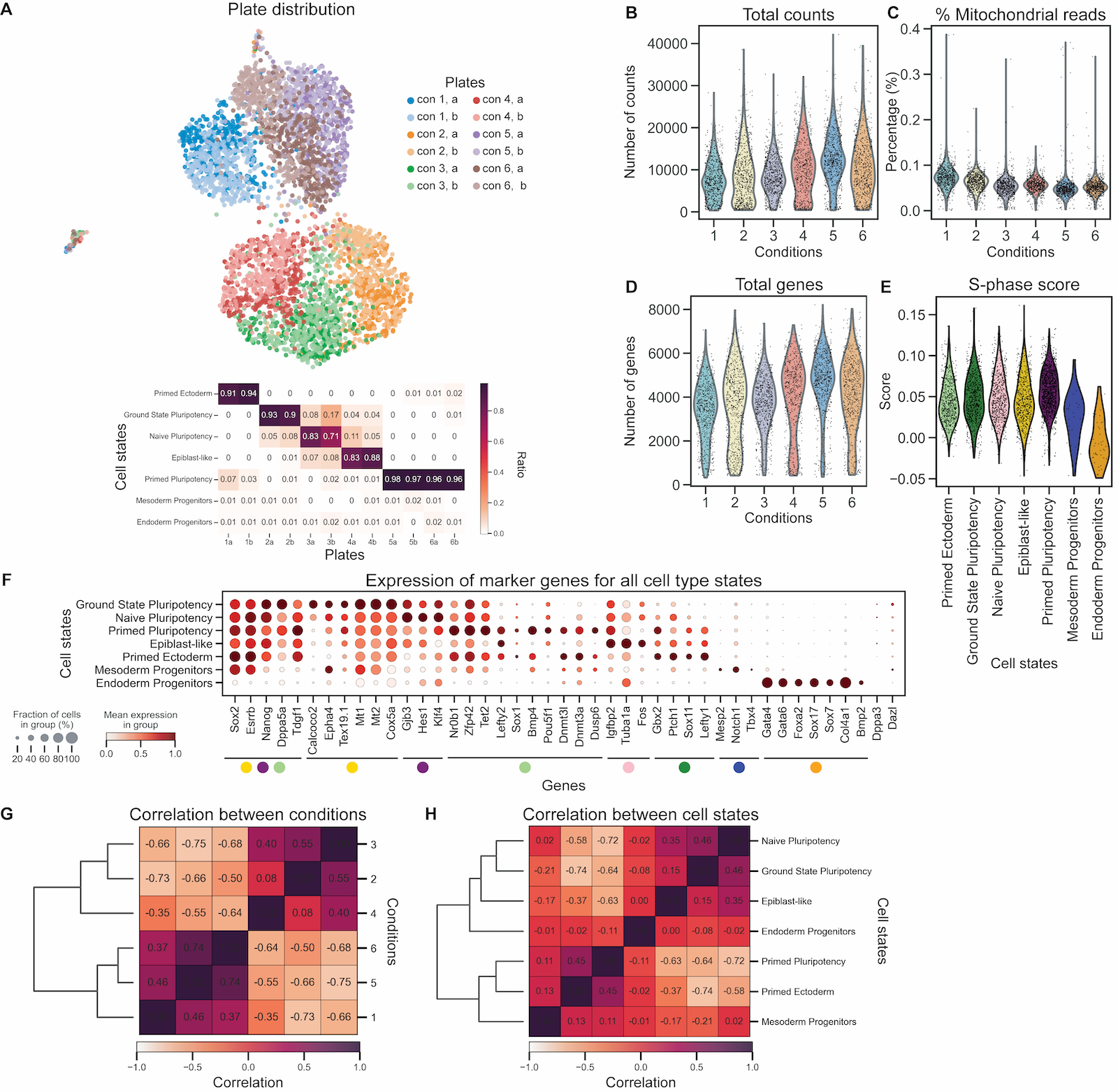

Supplement: S2 Fig — (A) UMAP coloured by plate distribution, insert: ratios of cell type states per plate. (B-D) Distribution of total count distribution (B), mitochondrial gene expression (C), and total gene count distribution (D) per cell. (E) S-phase scores per cell type. (F) Dot plot showing expression of marker genes used for determination of cell type states displayed in Fig 2B. Dot colour represents the mean expression of marker genes in specific groups, and dot size represents the fraction of cells within the group that show expression of the gene. (G-H) Correlation maps between conditions (G) and between cell type states (H). (TIF) [file pone.0317309.s002.tif]

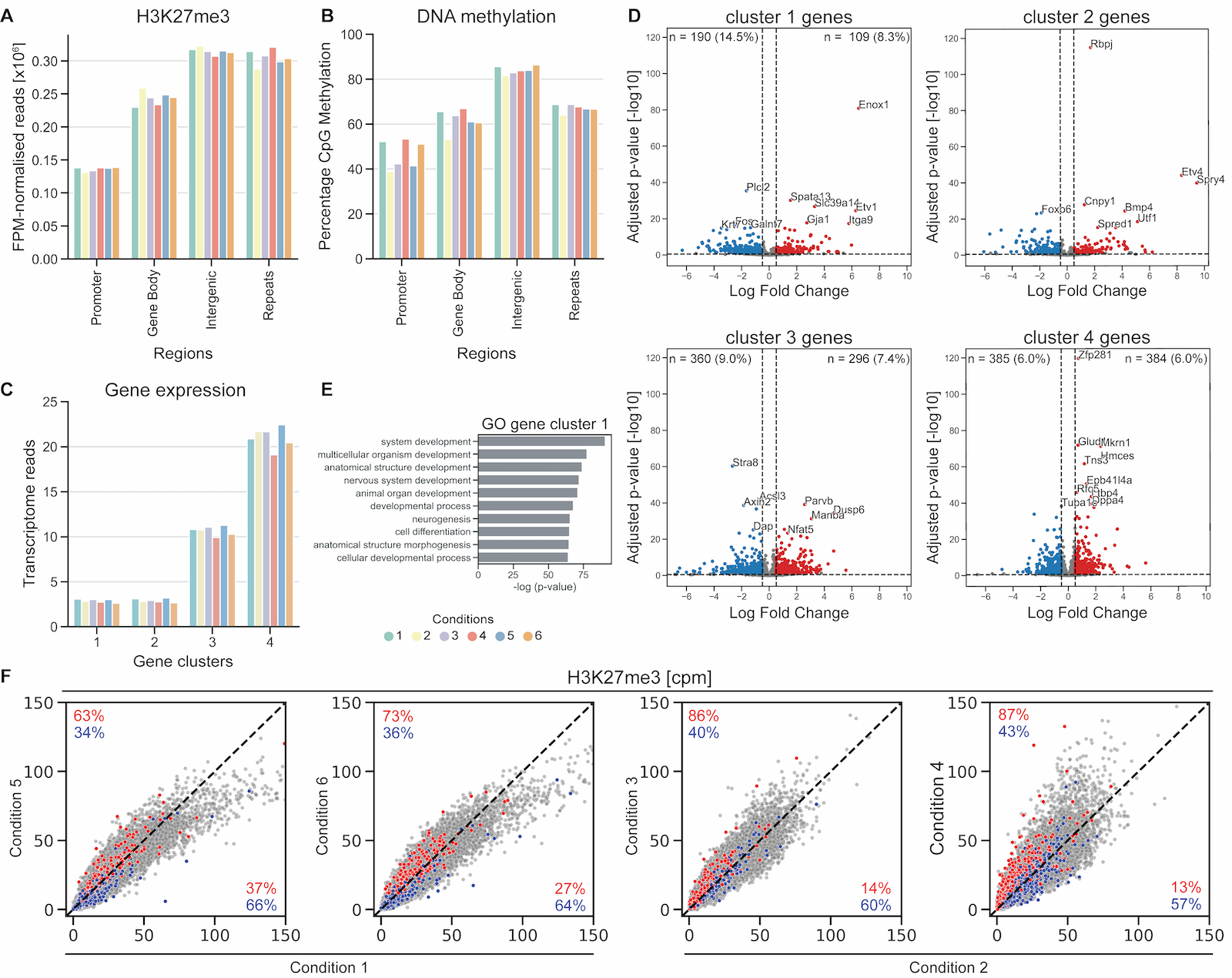

Supplement: S3 Fig — Normalised H3K27me3 abundance across different genomic regions for all six mESC pre-culture conditions tested. Counts are normalised for features per million (FPM), which takes into account the variable sequencing depth across samples and the variation in genome size of the different features. (B) Percentage of CpG methylation across different genomic regions for all six mESC pre-culture conditions tested. (C) Average level of transcriptome reads derived from scRNAseq dataset, grouped per pre-culture condition and gene cluster determined based on H3K27me3 data. (D) Volcano plots corresponding to Fig2C separated by identified gene clusters, indicating down regulated genes (left, blue) and upregulated genes (right, red) in ESLIF pre-culture conditions (conditions 1, 5, 6) compared to 2i pre-culture conditions (conditions 2, 3, 4). (E) Top 10 GO terms associated with gene cluster 1. (F) Scatterplots showing H3K27me3 counts (CPM normalised), found in 15 kb TSS windows comparing different pre-culture conditions. Coloured dots represent genes differentially expressed between indicated conditions, with red indicating genes upregulated and blue downregulated in the condition shown on the x-axis. Coloured numbers in the corners indicate the fraction of differentially expressed genes showing reduced H3K27me3 in the indicated condition. (TIF) [file pone.0317309.s003.tif]

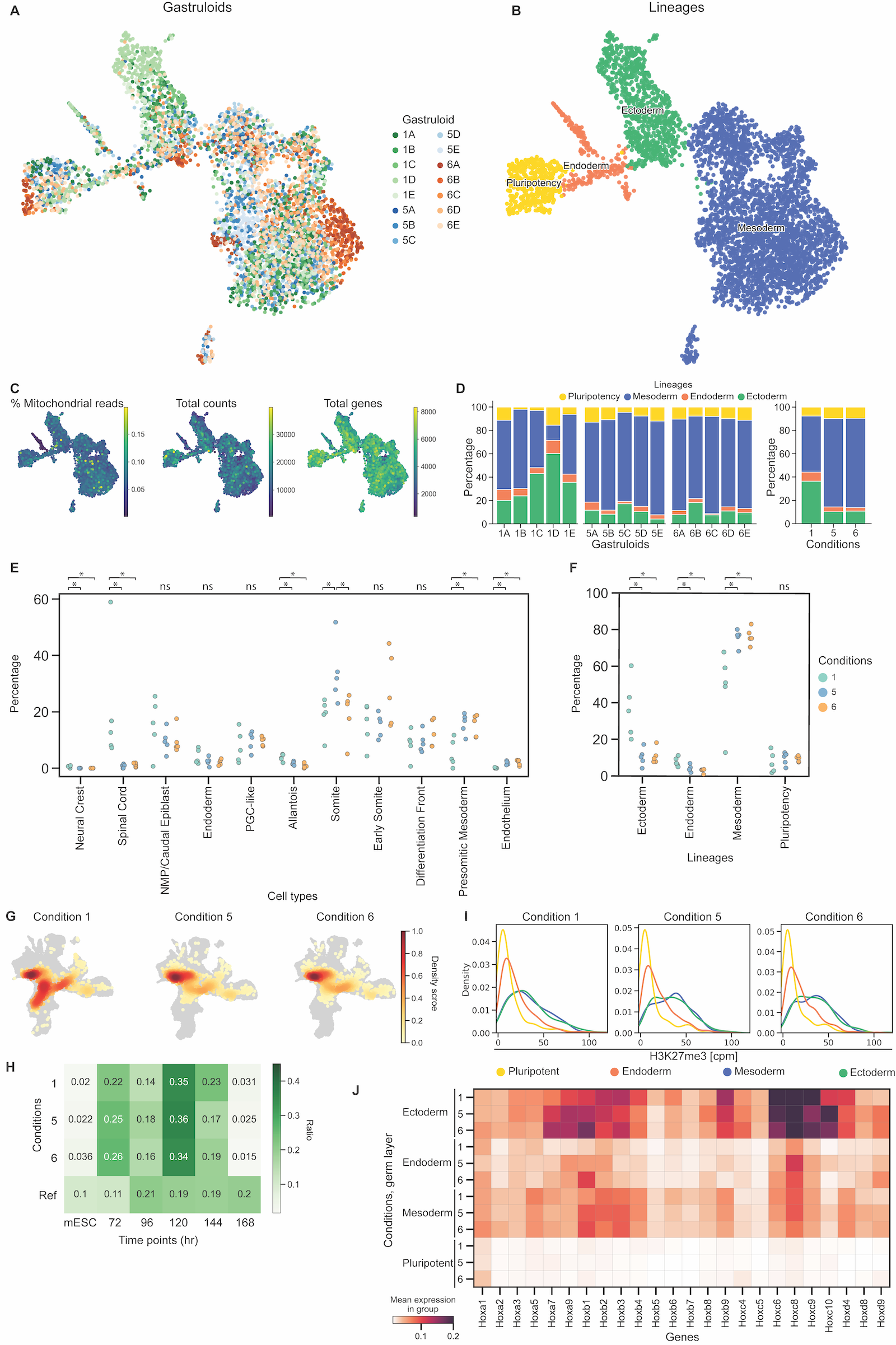

Supplement: S4 Fig — (A) Distribution of replicates across transcriptome UMAP. Five individual gastruloids were included for pre-culture conditions 1, 5, and 6. (B) Transcriptome UMAP separated by germ layers - endoderm (n = 230), mesoderm (n = 3,106), ectoderm (n = 882) - and pluripotency cells (n = 419). (C) Distribution of mitochondrial gene expression (left), total count distribution (middle), and total gene count distribution (right) per cell. (D) Ratio of germ layer contributions across 15 individually sampled gastruloids from condition 1 (1A-E), condition 5 (5A-E), and condition 6 (6A-E) (left) and between conditions 1, 5, and 6 (right). (E-F) Boxplots of cell type (E) and lineage (F) percentages per condition. Statistical significance between conditions was calculated with Wilcoxon rank-sum test with Bonferroni multiple testing correction. (G) Density plots of pre-culture dataset separated into conditions 1, 5, and 6, and the reference dataset. (H) Ratios of sampling times mapped onto the pre-culture dataset separated into conditions 1, 5, and 6, and the reference dataset. (I) Histograms showing H3K27me3 counts per TSS (CPM normalised), split by group of differentially expressed genes in gastruloids. (J) Heatmap of Hox gene expression across germ lines and conditions. (TIF) [file pone.0317309.s004.tif]
