# Supplementary material for: Stem cell culture conditions affect in vitro differentiation potential and mouse gastruloid formation
Source: PLoS One. 2025 Mar 26;20(3):e0317309. doi: 10.1371/journal.pone.0317309 (PMC11940422; doi:10.1371/journal.pone.0317309)
Supplement: Attachment — Submitted filename: Response_to_Reviewers_auresp_2.docx [file pone.0317309.s007.docx]

**Point-by-point response to the reviewer comments:**

**Reviewer #2:** I appreciate the authors' efforts in conducting some of the additional experiments and analyses I suggested, particularly the evaluation of the impact of distinct culture conditions on gastruloid morphology using different cell lines. These new results and the corresponding revisions have enhanced the manuscript, providing some clarity to the study. However, some issues are still unresolved and further work is required before the manuscript is ready for publication.

We thank the reviewer for their positive evaluation of the revised manuscript.

Main issues:

- The transcriptomic and epigenetic investigations remain limited to one cell line. Given the high variability found between cell lines in the initial culture conditions, it is important to extend these analyses to more cell lines and provide a thorough discussion of the results.

Indeed, the results of these proposed experiments would be highly interesting. Thanks to the previous reviewer comments and resulting manuscript revisions, we have included three cell lines in the first part of this study. This has generated important understanding on the effect of short-term pre-culture conditions on differentiation in different genetic backgrounds. While we would be curious to explore the transcriptome and epigenetic states of these different cell lines as described in the second part of this study for the B6 line, this would extend the contents of the manuscript far beyond our currently available time frame and resources.

In this discussion, the authors should also explain why longer periods of 2i treatment - recommended by laboratories such as that of Austin Smith to achieve a ‘tabula rasa’ state - were not considered for this study.

The work of Austin Smith and others regarding 2i treatment on mESCs has been highly influential and this state of long-term 2i culture has been extensively studied. Importantly, there are considerable differences between short- and long-term 2i culture in regards to the epigenetic and transcriptomic state of the cells. Upon growing gastruloids, we noticed that even a 24-hour pulse of 2i already considerably changed the morphology of the mESC starting culture and resulting gastruloids. For the purpose of this manuscript we therefore decided to focus on the effect of short-term pulses, with the hypothesis that subtle changes in pluripotency state affect downstream differentiation programs and would aid in defining a protocol with increased stability and reproducibility. To our knowledge, the use of short-term pulses of 2i has been less studied than the clearly defined long-term 2i state. We have added this statement to the discussion, p19, lines 512-514:

*Since the impact of such long term 2i treatment on ES cell differentiation state and gastruloid formation was already thoroughly studied, in this work we focused on shorter 2i pulses to adjust and synchronise their cellular states.*

The manuscript still lacks sufficient evidence showing whether the different culture conditions alter the cell state of the gastruloids at 48 hours (before CHIR treatment), and if this causes the observed differences at 120 hours. It is critical to provide this data to interpret the CHIR timing shift correctly. For instance, is symmetry breaking delayed when 2i is used? The rationale for altering the CHIR treatment timing needs to be clearly explained, with evidence.

After careful re-evaluation of the paragraph on p.12, lines 298-304, we think we may have inadvertently caused some confusion. The main and intended message of this paragraph is that in Supp Fig G-I we found no significant evidence that a shift in timing of the Chiron pulse improved gastruloid elongation, with the exception of condition 3. While this was important to control, all other experiments in the manuscript therefore were performed with the standard Chiron pulse (48-72 hours). We apologise that we did not formulate our description and conclusions from these experiments more clearly and have made the following textual changes to reflect this:

p.12 lines 298-304 previously read: “*Because the timing of the chiron pulse could influence gastruloid formation, we subjected all conditions additionally to a delayed chiron pulse from 72 h - 96 h AA, and assessed them at 120h or 144 h AA (Fig S1G-I). While gastruloids from most pre-culture conditions show a lower AR following the delayed chiron pulse, condition 3 cells display a significant increase in AR after a delayed pulse and delayed read-out (Figs S1H-I). Therefore, we subjected condition 3 pre-culture cells to a 72 h - 96 h AA chiron pulse and read-out at 144 h AA, while for all other pre-culture mESCs we used the default gastruloid protocol.*”

In the current, revised version, we now included: “*Because the altered pluripotency state of the different pre-culture conditions could have an effect on symmetry breaking during the gastruloid protocol,* we *subjected all conditions additionally to a delayed chiron pulse from 72 h - 96 h AA, and assessed them at 120h or 144 h AA (Fig S1G-I). We only observed a significant increase of the aspect ratio for pre-culture condition 3 with a 24h delay of the chiron pulse and read-out (Figs S1H-I). Therefore, we subjected condition 3 pre-culture cells to a 72 h - 96 h AA chiron pulse and read-out at 144 h AA, while for all other pre-culture mESCs we used the default gastruloid protocol.*”

As previously suggested, stainings for key primitive streak markers like Brachyury between 48 and 72 hours would be a solution.

We thank the reviewer for this comment, which indeed helps further interpretation of our findings. We have added the proposed experimental data in Fig S1J-K and describe the results on p12, lines 304-309:

*To investigate what causes this difference between pre-cultures, we assessed the time point of Brachyury (T) induction and polarisation between pre-cultures (Fig S1J-K). We found that while condition 1 results in polarised structures at 72h AA, conditions 2 and 3 display a delay in T induction and polarisation. Condition 4 pre-cultured gastruloids display similar symmetry breaking as compared to condition 1, but with a lower overall expression of T. At 96h AA, all pre-culture conditions result in elongated structures with polarised expression of T.*

Minor issues:

- It should be visually clear which results in Figure 1 correspond to which cell line. The same applies to the new figures.

We have clarified the cell lines used within figure panels 1A’, 1D-E, and S1D-I. Figures 2, 3, S2-4 were generated with the B6/129 mESC cell line only, and we have clarified this in the titles of the figure descriptions. This is also specified in the text, p.12 lines 295-297: *We decided to study the effect of all pre-culture conditions in more detail in the B6 cell line to see if other conditions could lead to more consistent results across replicates.*

- The data suggesting the involvement of Shh signalling (“ectodermal priming signature with the expression of Gibx2, Ptch1, Sox11, and Lefty1, indicative of Sonic Hedgehog (Shh) signalling”) is insufficient and lacks appropriate references. Additionally, the role of this signalling in pluripotency should be discussed.

We thank the reviewer for this comment, and apologize for the lack of appropriate references for this statement. This statement is now rewritten for clarity (p.14, lines 361-363: *“...and condition 1 mESCs show expression of factors associated with Sonic Hedgehog (Shh) signalling, such as Gbx2, Ptch1, Sox11, and Lefty1, indicative of ectodermal priming (Fig S2F)”).* Appropriate references (1) identifying Gbx2, Ptch1, Sox11, and Lefty1 as Shh pathway components and (2) implicating Shh signalling in ectodermal specification are now provided.

- The authors’ annotation of the different cell types and pluripotency states has improved but still needs refinement. For example, is Tbx4 a mesodermal marker? I suggest the authors review each marker mentioned in the text and provide proper references.

We have provided references for each (list of) used marker genes. We apologise for the confusion surrounding Tbx4, indeed it has been used as a marker gene for allantoic mesoderm (see references 15, 53) and we have now specified this accordingly in the manuscript.

- The issue of potential heterochrony should be resolved by including a heatmap showing the expression of Hox genes across the different conditions.

We have included the requested figure panel in S4J.

- The authors should lower their claims regarding the novelty of their screening approach as similar methodologies have been published already by other laboratories (e.g. Lutolf’s lab).

We now highlight previous work using screening approaches to improve *in vitro* differentiation protocols in the discussion (p20, lines 537-539):

*Importantly, building upon previous work to systematically improve gastruloid efficiency and complexity (71,72), we present an unbiased screening approach to assess the efficiency of gastruloid formation.*
