# Supplementary material for: Stem cell culture conditions affect in vitro differentiation potential and mouse gastruloid formation
Source: PLoS One. 2025 Mar 26;20(3):e0317309. doi: 10.1371/journal.pone.0317309 (PMC11940422; doi:10.1371/journal.pone.0317309)
Supplement: Attachment — Submitted filename: Response to Reviewers.docx [file pone.0317309.s006.docx]

Dear Dr. Pina,

Please find attached the revised version of our manuscript “Stem cell culture conditions affect *in vitro* differentiation potential and mouse gastruloid formation” for your reconsideration. We thank the editor and reviewers for their positive and constructive feedback on our manuscript.

To address the concerns of the reviewers and improve the manuscript, we have now included data from 2 additional cell lines derived from a different genetic background to evaluate consistency of our findings across cell lines. Importantly, the IB10 wild-type line included is widely used by other laboratories for generating gastruloids, and we now collaborated with two of these labs to produce this additional data, as reflected by the updated author list. The TR line is included as a non-wild-type line, which due to the three germ layer reporters (including Brachyury) is a highly valuable addition to our manuscript to assess early differentiation of cells in the gastruloids.
We have expanded both the details of the method description and the data analysis to ensure reproducibility of the experiments. In addition, we have updated our code and data accessibility to include GEO accession codes to all data, and provide annotated pipelines and notebooks of the performed analyses.
Lastly, we included both FACS analysis of germ layer-specific marker gene expression and pseudotime analysis of the gastruloid single-cell RNA sequencing data matched to a time-course dataset, to ensure the absence of a general developmental delay between pre-culture conditions. While the pseudotime analysis provides an internal control of developmental timing in the datasets included in this manuscript, the FACS approach provides an accessible tool to control for this aspect in more comprehensive future experiments. More detailed explanations can be found below, in a point-by-point response to the reviewer’s comments. We have also attached our updated Acknowledgements and Funding Information sections on the next page, in line with the style requirements.

We adjusted the indicated sections to adhere to the requirements for submission as listed by the editor in the decision letter. Altogether, we think that the new data provides valuable additional insights into how stem cell culture conditions affect in vitro differentiation potential and mouse gastruloid formation, thereby strengthening and supplementing our study. The manuscript has been revised accordingly, the figure and page numbers below refer to the new manuscript. After implementing all the issues of the reviewers that you specifically highlighted to us as critical, as well as most other suggestions of the reviewers, we hope that our study is now judged acceptable for publication in PLOS ONE.

Kind regards,

Marloes Blotenburg and Peter Zeller, corresponding authors

On behalf of all authors

**Point-by-point response to the reviewer comments:**

**Reviewer #1:** In the manuscript entitled “Stem cell culture conditions affect in vitro differentiation potential and efficiency of mouse gastruloid development” Blotenburg and colleagues confirmed and extended previous observations that the cell culture conditions, and specifically the pluripotency state of the stem cell population, affect gastruloid development in term of morphology, elongation, and cell type composition. By performing ScRNA-seq analysis of mESCs and gastruloids the authors identify the pathways that control the efficiency of gastruloid formation. The results are interesting and provide insights into the complexity of stem cell plasticity. However, in my opinion, there are important points that need to be addressed prior to publication.

 We thank the reviewer for their very positive evaluation of our manuscript.

 Major points:

The impact of different culture conditions on gastruloids formation have been previously reported. Unfortunately, the authors incorrectly cite the relevant literature. For instance, Cermola et al (Stem Cell Report 2021, ref.31) reported the effect of ESCs grown in different culture conditions//pluripotency state including but not limited to i) DMEM/FBS 15%/LIF, ii) N2B27 2i/LIF, and FGF/Activin -induced EpiLC and EpiSCs on gastruloids formation. They demonstrated that culture conditions significantly impact gastruloid formation, affecting i) cell aggregation (size of cell aggregates at 48 hours), ii) elongation (proportion of correctly elongated gastruloids), and iii) morphology (presence of aberrant gastruloids/multiple protrusions).
 The authors should discuss their findings more thoroughly in the context of the current literature and properly cite the relevant papers. This will not diminish the significance of their studies.

We are happy that the reviewer appreciates the relevance of the presented work. We have adjusted and expanded our text to make sure existing literature is accurately cited and discussed. We have re-written the introduction to more carefully reference existing literature and included the reference of Anlas et al (Methods Mol Biol, 2021, PMID 33340359) where the impact of 2i pre-culture on gastruloid formation was reported on p10 line 239-240 and on p17 line 470. We have more accurately cited Cermola et al., and expanded the discussion on p17, lines 468-474 to include that different culture conditions between studies can also affect the reported gastruloid formation:

*“In addition, another study using E14 mESCs reported increased efficiency of elongation after mESCs were maintained in 2i medium, and some cell lines require a 2i pulse for gastruloid formation (51, 53). It is therefore plausible that different cell lines require alternative pre-treatments to achieve the same receptive state as required for symmetry breaking during gastruloid formation. Additional experimental variations between studies, such as alterations in media composition and variations in the cell culture protocol, could also influence the reported differences.”*

Materials and Methods
 - Pag. 4, line 84: PBS0 and TrypLE are not defined. Please specify
 - Pag. 5, lines 92-93: the concentration of 2i inhibitors (CHIR and PD) are expressed as %, but it is not clear % of what mass/vol?. Please clarify or indicate the molar concentration.
 - Pag. 7, line 147: the sentence ‘Cells with either less than 500 reads and 100 genes detected in less than 2 cells were filtered out’. Please rephrase or clarify

This information was indeed missing in the methods description and is now added on page 5 line 108-109 (PBS0 and TrypLE) and p5 line 111-113 (CHIR and PD). Because two additional cell lines were included, the methods sections “cell culture and gastruloid generation” and “bright-field microscopy and analysis” (p4-6) were expanded. The sentence “Cells with either less than 500 reads and 100 genes detected in less than 2 cells were filtered out” was rephrased to “Cells with less than 500 reads or less than 100 genes detected were filtered out.” on p8, line 183.

 Results

Pag. 8 lines 195-196: the authors claim that ..”pluripotency states in between naïve and ground state can be produced with short-term pulses of 2i and ESLIF”. However, this conclusion lacks experimental evidence. The authors should provide at least some characterization of the cells defined as “intermediate” states of pluripotency (conditions named 3, 4, 5 and 6) between conditions 1 and 2.

We agree that the microscopy-focused analysis in Fig1 is indeed not sufficient to prove the existence of intermediate states of pluripotency, rather than different mixtures of defined states. Therefore, we now expanded the single-cell differential gene expression and H3K27me3 analysis in Figs 2D-G, S3F and S4I. Together, these analyses show pre-culture specific transcription and chromatin states, also for intermediate states tested. Especially the single-cell data supports the statement of different cell states rather than different state mixtures.

 Figure 1, pre-culture conditions, including the number of cells plated/density, the type of plates and coating used, are not specified.

We thank the reviewer for this comment. We now added the missing details about plates and cell densities used to the methods section on p4, line 99 (“gelatin-coated 6-well plate cell culture dishes”) and p5 line 104 (“Cells were split every second day at 80% density“). We also elaborated on the culture conditions used before gastruloid aggregation on p5, lines 118-119: “two days before aggregation, cells were plated in a series of 1:10 to 1:3 dilution, and at the time-point of aggregation the cells with 80% confluency were chosen”

Figure 1, panel A’. At the high cell density, assessing the effect of culture conditions on cell colony morphology seems difficult to evaluate/quantify. Therefore, the phenotype-based analysis of the intermediate states proposed here lacks convincing evidence. On the other hand, a molecular-based approach, i.e. the identification of specific markers for the 6 conditions, for instance based on data from the RNAseq analysis, seems not sufficient per se (see below).

We thank the reviewer for this comment. From what we understand from this remark, it seems this comment is based on a misperception due to the low magnification of the shown images. We now changed the images in Fig 1A’ for higher magnification examples that allow a clearer recognition of condition-specific colony morphologies. We also provide representative images of the newly included cell lines in Fig S1A. We have addressed the suggestion for the molecular-based approach in the points below, where we explain the expanded scRNA-seq analysis of the six pre-culture conditions in mESCs presented in Fig 2D-G and Fig S3D. We further elaborate on the differences in gene expression between all conditions.

Pag. 9 line 204. …”cells from all pre-culture conditions except ‘condition 2’ generate gastruloids efficiently”. The 2i medium is routinely used to capture naïve pluripotency, and so, the observation that ESCs grown in 2i medium (condition 2) are unable to elongate is unexpected. It is likely that 2i ESCs are ‘locked” in the naïve pluripotent state and that this eventually delay/prevent exit from pluripotency and symmetry breaking. Indeed, condition 5, that is 48h in LIF medium is sufficient to rescue the elongation efficiency (see also Discussion section below). Please, discuss.

Indeed, the B6 cell line shows a decreased axis ratio (AR) after 2i incubation, which contradicts previous observations mentioned by the reviewer. We have now added two more cell lines from a different genetic background, which show different dynamics. As a matter of fact, the IB10 line shows an increased AR after 2i culture. We therefore show that the effect of pre-culture conditions on gastruloid formation (and more specifically their AR) is cell line-dependent. We agree that it is possible that 2i ESCs from the B6 line are ‘locked in’ a naive pluripotent state, which could delay their exit from pluripotency and thereby differentiation. We have included an additional analysis in Figures 3F-H and S4G-H to address the question of developmental delay between pre-culture conditions, based on a suggestion by reviewer 2 (see discussion on heterochrony below). In this analysis, we did not observe evidence of developmental delay between the conditions tested, but it should be pointed out that we could only compare pre-culture conditions 1, 5, and 6. While we are not able to conclude whether condition 2 would result in a developmental delay in the B6 line, the results of this analysis underline the reviewer’s comment that in condition 5, a 48h pulse in ESLIF medium is sufficient to result in timely differentiation. We further hypothesise that it is possible that the ‘default’ pluripotency state differs per cell line and genetic background, and this in part explains their different responses to the pre-culture conditions tested as we now show in our manuscript. We have now included this in the discussion on page 17, lines 463-474:

*“Here, we show that by modulating the mESC state through pre-culture media composition, the elongation length and reproducibility of gastruloid formation is affected. We first tested three different cell lines of various genetic backgrounds and noticed that the pre-culture condition resulting in the highest AR differs per cell line, highlighting the need for cell line-specific optimisation of the gastruloid protocol. While the B6 background yielded the lowest AR of gastruloids after 2i culture, this was not replicated in the TR and IB10 cell lines. In addition, another study using E14 mESCs reported increased efficiency of elongation after mESCs were maintained in 2i medium, and some cell lines require a 2i pulse for gastruloid formation. It is therefore plausible that different cell lines require alternative pre-treatments to achieve the same receptive state as required for symmetry breaking during gastruloid formation. Additional experimental variations between studies, such as alterations in media composition and variations in the cell culture protocol, could also influence the reported differences.”*

Figure 1, the values of the elongation index are overall lower than those previously reported by different laboratories (2.5 or higher), and the abnormal value (see Fig.1, panel D, conditions 3, 4, 6 replicate B) appears to correspond to the diameter rather than the length. Indeed, the diameter is larger than expected (100-200 microM). Likely, it is due to the use of the largest circle (from Max Inscribed Circles Fiji plug-in) as the gastruloid diameter.

To avoid a potential miscalculation due to the usage of the largest circle, we improved the analysis using the MOrgAna software, as this estimates the length and width of gastruloids more accurately than the approach of using the diameter of the largest circle. Of note, this change in indexing of the gastruloids did not yield significant changes to the elongation index, confirming that the presented quantification does represent the imaging data. Having now included two additional cell lines we see larger elongation indexes for the IB10 cell line that are more similar to previous reports. We therefore conclude that the observed differences are cell line-specific.

Pag. 10 line 242-243, ScRNAseq analysis (Figure 2) revealed that ESCs even if grown in 6 different conditions, display only two different transcriptional and epigenetic signatures. These results raised some issues:
 a.- What about the intermediates states of pluripotency. Is the ScRNAseq analysis able to discriminate the cells grown in the 6 conditions? The cells pre-grown in the 6 different conditions display significant differences in their transcriptome? Please clarify

We agree that the intermediate states of pluripotency presented in this manuscript were not highlighted sufficiently, and therefore included a more detailed analysis of the condition-specific differences in Fig 2D-G, including differentially expressed genes and a biological process enrichment analysis (p13, lines 342-349). We also expanded the analysis of H3K27me3 coverage in these conditions in figure S3F (p14-15, lines 382-385). Based on the differential gene expression analysis (Fig 2D-G) we now see that condition 1 is primed towards an ectoderm fate, but conditions 5 and 6 are not. Down-regulated genes in condition 2 compared to conditions 3 and 4 are enriched for GO terms of organismal development, indicating that conditions 3 and 4 display a less naive stage of pluripotency than condition 2. The expanded H3K27me3 analysis shows a loss of H3K27me3 on transcriptionally upregulated genes of the same condition.

b. Is the transcriptome profile relevant for the gastruloid formation efficiency?. For instance, cells from conditions 1, 5, and 6 seems to segregate together from the seq analysis but display a completely different gastruloid formation efficiency (Figure 1).
It seems that the presence of LIF for the last 24 hour (compare conditions 1, 5, and 6) is sufficient to induce a similar signature/cell identity (transcriptome and epigenetic signature).

We thank the reviewer for raising this interesting issue. As shown in figures 2D-G and S2F that we now expanded based on this comment, we can see ectoderm-specific genes getting expressed in condition 1 mESCs. This is consistent with the scRNAseq dataset of gastruloids (figure 3), where we see a higher ratio of ectodermal cells in condition 1 gastruloids. This indicates that the transcriptome of the mESC might be a predictive factor. Beyond this, our analysis could not identify further germ layer commitment by any of the conditions. This could potentially relate to the time points of the analysis, since we compare mESCs with 120 h AA gastruloids and this study does not include time points in between these states. It is possible that these subtle differences observed in mESCs are enlarged around the time point of WNT activation.

 Pag. 13 line 323 to Pag. 14 line 346, ScRNAseq analysis (Figure 3) revealed that gastruloids generated with cells grown under different conditions (1, 5, and 6), have a different lineage compositions. These interesting results raise some issues:
 a.- Is the composition of the gastruloids analyzed here (mainly condition in line with that reported in the literature with respect to types/number of cell lines identified, etc.?

The results text was adjusted to include a comparison of the observed differentiation biases with relevant literature. Cell types were annotated based on established marker genes shown in existing literature (page 16, lines 420-421). We elaborate on the comparison of cell type composition reported in Rosen et al., 2022, in the discussion on p18, lines 481-488:

*“The gastruloid protocol was also reported to result in inter-gastruloid heterogeneity, with individual gastruloids being biased towards either an ectoderm or a mesoderm fate (22). This bias in specification was caused by differences in response strength to the Wnt-activation pulse. To take inter-gastruloid variability into account, we profiled 5 individual gastruloids per pre-culture and compared lineage contributions between replicates and between conditions. We did not observe similar inter-gastruloid heterogeneity in this dataset. Future studies are required to shed further light on how cellular heterogeneity in responsiveness to extracellular signals contributes to lineage decisions.”*

b.- the high fraction of mesoderm in gastruloids derived from conditions 5 and 6 correlates with the induction of mesodermal markers in ESCs grown under these conditions?

In the new figure panels Fig 2D-E we indeed see the mesodermal gene Tbx3 as differentially expressed. However, more likely than c5/c6 being primed for mesoderm is that condition 1 is primed for ectoderm instead, indicated by marker gene expression in Fig S2. This is also indicated by condition 1-specific Shh signalling. The text was adjusted to address this question (p13 lines 342-349, p15-16 lines 410-414, p16 422-425).

 Discussion
 Pag. 14, lines 362-364. …”While in our study 2i grown mESCs yielded the lowest efficiency of gastruloid elongation, another study using E14 mESCs reported increased efficiency of elongation after mESCs were maintained in 2i medium. (31)”. This is not correct. In the paper by Cermola et al. (ref 31 ms), mESCs were maintained (passaged) in DMEM/FBS 15%/LIF not in 2i. Then, to generate gastruloids, mESCs were seeded at a low density (300 cells/cm²) on gelatin-coated plates and cultured in N2B27 2i/LIF for 5 days. It is important to note that the 2i medium also includes LIF (2iLIF). Under these culture conditions, 90%–95% of ESC colonies show a domed round-domed shaped phenotype. Besides the difference in the culture conditions, other technical differences in the protocol need to be consider:
 1) mESCs were dissociated with a milder accutase treatment, and not trypsin
 2) The protocol includes a FACS sorting step to eliminate dead cells and cellular debris prior to aggregation.
 Thus, the culture conditions and the protocols used in the paper by Cermola et al. (ref. 31) and those reported herein are different and should be considered. These important differences should be addressed and discussed, and the sentence should be rephrased accordingly.

We thank the reviewer for this clarification and have adjusted the discussion to report experimental differences more accurately (page 17, lines 473-474)

**Reviewer #2:** The study of Blotenburg et al. addresses whether stem cell culture conditions, particularly the introduction of 2i, affect the development of mouse gastruloids and whether an optimised mESC pluripotency state would provide more consistent results. These are relevant questions because the gastruloid model system is widely used and, contrary to the 3D/N2B27 part, 2D culture conditions lack a defined protocol and have been shown to vary substantially (sometimes even in the same study).

We thank the reviewer for seeing the impact of our study.

Throughout the manuscript the authors present results suggesting that modulation of the pluripotency state of mESCs significantly affects the phenotype (morphology), transcriptome and epigenome of gastruloids, ultimately impacting the specification of certain cell fates. Although these results are interesting, they were obtained from only one mouse cell line. Also, the same occurs for the optimisation work carried out to improve the reproducibility/consistency of the gastruloid system. Checking the ‘universality’ of their findings in at least two more mESCs (also used by other labs working with gastruloids) would significantly improve the work and its significance to the community.

We have added two more cell lines (TR and IB10) from a different genetic background to the study. The IB10 is a wild-type line widely used by other laboratories for producing gastruloids (o.a. by many laboratories in the Netherlands, some of which we collaborate with as now reflected in the new author list of the paper), and the TR line is included as a non-wild-type line to indicate the effect reporters might have on cell line performance. Indeed, the exact impact of individual pre-culture conditions appears to be cell line-specific. A clear example is the 2i-only pre-culture (condition 2), which produces gastruloids with the lowest aspect ratio using the B6 line but displays a higher AR in IB10 gastruloids, consistent with current literature. For all three cell lines, the pre-culture conditions tested have significant impact on symmetry breaking and germ layer contribution. Importantly, the studies with the TR and IB10 cells were performed in a different lab, underscoring the universality of this study.

Another aspect that requires further work regards the control of cell number during 2D culture. According to the manuscript, cells were split every second day in a 1:5 ratio during the modulation of their pluripotency state. Did the authors examine whether cell number was kept stable across the different conditions? If the numbers changed, they should check whether seeding density alone affects the pluripotency state and, further down, gastruloid formation. It is important to isolate/control the seeding density variable during these experiments to understand the 2i effects more accurately.

We expanded the methods description to make this point more clear for all included cell lines.. During cell maintenance, a density of 80% was achieved before splitting the cells 1:5 (p5, line 104: “Cells were split every second day at 80% density” and p5, lines 106-107: “and replating the pellet 1:5 (B6) or 1:10 (IB10, TR) on gelatinised plates.”). During the last split before gastruloid aggregation, mESCs were passaged at five different densities. At the moment of gastruloid aggregation, the dilution that reached 80% confluency was used. This is now specified on p5, lines 118-119: “two days before aggregation, cells were plated in a series of 1:10 to 1:3 dilution, and at the time-point of aggregation the cells with 80% confluency were chosen.”

After modulating the 2D culture conditions, Blotenburg et al. developed mouse gastruloids according to the standard protocol and performed a detailed morphological analysis. Here, it would be nice if the authors pointed out the morphological differences they see in the gastruloid images of Figure S1A (e.g. “ridge in the centre and protrusions at the posterior end”).

While these morphological differences are very clear by eye (also see example pictures), quantifying them in an automated and robust fashion proved to be challenging, also with the new quantification method that we used based on the MOrgAna software. We therefore removed the statement in question.

The authors then tested the effects of a temporal shift in the CHIR treatment in the various culture conditions. However, it is not possible to correctly interpret this experiment without any Brachyury stainings at 48, 72 and 96h.

Indeed, Brachyury stainings at these time points would be useful to determine the symmetry breaking of gastruloids in different conditions tested. We have included data of a triple reporter (TR) cell line, using Brachyury, Mt1 and Sox17 as its markers. At 120 h AA, we observe that in this cell line, Brachyury expression is higher in pre-culture condition 2 (2i) than condition 1 (serum). For gastruloid elongation, we decided in the current manuscript to quantify this by defining the minor and major axis of the gastruloids, and use these to calculate the aspect ratio (AR). We use the AR as a proxy of symmetry breaking and early differentiation, where any AR > 1 indicates an elongated structure. In addition, to confirm that the selected conditions are comparable, we have also included an analysis where we integrate the single-cell RNA seq data from formed gastruloids (Figures 3, S4) with a published time course dataset (see discussion on heterochrony below). Here, we show that cells from all analysed conditions are comparable in terms of developmental timing. We suggest that these analyses together provide sufficient information regarding the timing of symmetry breaking in the conditions tested, even without the inclusion of brachyury stainings along a time-course.

 Also, Blotenburg et al. should perform the read-out of condition 2 at 144h; there is published data indicating that gastruloids whose cells have been treated with 2i only achieve their maximum elongation at that time (e.g. Beccari et al., 2018).

We acknowledge that for conditions 1 and 2, these later time points are missing in the presented dataset. Unfortunately, we were not able to repeat these experiments. To better understand relative developmental timing between conditions, we have included additional analyses regarding the time point of symmetry breaking in gastruloids of different conditions, using the calculated aspect ratio and a new analysis where we compare the single-cell RNA-sequencing data of gastruloids to a time course dataset (see the comment above).
Based on the reviewer’s suggestion, we have additionally included data from two different cell lines derived from a distinct genetic background. In the IB10 cell line now included, we observe that condition 2 results in the highest aspect ratio at 120 h AA. We therefore hypothesise that the observed effects of condition 2 in the B6 cell line are cell line-dependent rather than time point-dependent. We have further elaborated on the effect of 2i culture in the different cell lines above, in an earlier comment of the reviewer.

Finally, the authors should also revise terms like “ ‘perceptiveness’ to the CHIR treatment” and explain the meaning of “ ‘efficient’ gastruloid formation”.

We now have clarified our statements to indicate the effect of a delayed chiron pulse on gastruloid formation (“perceptiveness to chiron”), and specify that “efficient gastruloid formation” is quantified by a high aspect ratio (AR).

In the last part of the manuscript, the authors address the transcriptome and epigenome comparison they did for the different culture conditions, both before and post gastruloid formation, and conclude that there are significant differences in their molecular signatures. Here the authors must revise their annotations and try to be more accurate in defining the different pluripotency states and cell types present in both the pre-culture and gastruloid datasets. For defining the different pluripotent cell states, the authors should use terminology, supported by gene references, that have already been published in other studies (e.g. Nichols and Smith, 2009, Smith 2017, Morgani et al., 2017 and Cermola et al., 2021). Regarding the gastruloid data, Blotenburg et al. should expand the number of genes used for annotation (e.g. Tbx1, Tcf15, Sox17, Noto, Pax6, Otx2, etc.) and use correct anatomical/embryological terminology (e.g. ‘Caudal epiblast’ instead of ‘Tailbud’, ‘Spinal cord’ instead of ‘Neurons’, etc.).

We have revised our annotations, both of the mESC as well as of the gastruloid scRNA-seq datasets, using the publications and marker genes suggested by the reviewer, and have referenced the publications used. Annotations of cell states and cell types are adapted accordingly in figures 2B and 3B, and the marker gene panels are adapted and expanded in figures S2F and 3C.

Importantly, the authors also need to look at genes that can provide some information about developmental time as it is critical to discard heterochrony; UMAPs showing the integration of the different gastruloid datasets with that of embryos would be very helpful as well.

We have addressed the issue of heterochrony by comparing the gastruloid data to a time-course dataset of the same cell line in figures 3F-H and S4G-H. This reference dataset profiles gastruloids from 72 h AA until 168 h AA, with 24-hour time intervals. We found that the three pre-culture conditions tested in this dataset fall at the same point of pseudotime in the reference dataset, and therefore found no indication that the pre-culture conditions tested here cause a shift in developmental timing during gastruloid differentiation. This integration has also allowed us to determine the lineage composition of gastruloids derived from these pre-cultures in an independent manner, which confirmed an increase in ectodermal contribution in pre-culture condition 1 gastruloids.

Additionally, the authors should include in the materials and methods section information about the number of cells analysed per condition during the previous single cell experiments.

We have specified the requested information in the materials and methods section (p6-7, lines 154-157: “Dissociated single cells were sorted into two 384-well plates for each pre-culture mESC condition, resulting in a total of 768 cells per condition. For each individual gastruloid, one 384-well plate was sorted and processed, and 5 gastruloids were sorted per condition.”).

One of the key results of this manuscript is the finding that modulation of pre-culture conditions, with the addition of 2i, seems to have a strong effect later on cell fate specification during gastruloid formation, particularly in the amount of neural and mesodermal tissues. This finding contrasts with what is reported in the preprint of Rosen et al., 2022, which also finds a bias in the amount of neural and mesodermal cells but links it to inter-gastruloid heterogeneity; Blotenburg et al. should discuss this issue.

As indicated by the reviewer, the comprehensive study of Rosen et al., 2022 is of particular relevance for our findings. We have now addressed this study in the discussion on p18, lines 481-488:

*“The gastruloid protocol was also reported to result in inter-gastruloid heterogeneity, with individual gastruloids being biased towards either an ectoderm or a mesoderm fate (22). This bias in specification was caused by differences in response strength to the Wnt-activation pulse. To take inter-gastruloid variability into account, we profiled 5 individual gastruloids per pre-culture and compared lineage contributions between replicates and between conditions. We did not observe similar inter-gastruloid heterogeneity in this dataset. Future studies are required to shed further light on how cellular heterogeneity in responsiveness to extracellular signals contributes to lineage decisions.”*

Importantly, both the study by Rosen et al and our study include replicates of individual gastruloids, allowing the study of inter-gastruloid heterogeneity. Our study includes 5 single-gastruloid replicates for each condition, and while we agree that a higher number of replicates would be desirable, this was not achievable in our current study due to experimental limitations. We now included in the discussion (p17-18, lines 470-476 in the revised manuscript) that the degree of inter-gastruloid heterogeneity might be influenced by the cell line, protocol and experimental conditions (including pre-culture conditions) used.

Overall, the main question addressed in this manuscript is pertinent and the experiments are interesting. However, there is a considerable amount of work to be done in order for the results to be accurate and significant to the community.

We thank the reviewer for their critical feedback and useful suggestions which we have tried to address carefully and thoroughly, among others by including a large number of new results from different cell lines and markers (within the TR line) to further substantiate our findings. We believe it has improved our manuscript and hope the changes have highlighted the relevance of our study for the community.
